# Lay Health Trainers Supporting Self-Management amongst Those with Low Heath Literacy and Diabetes: Lessons from a Mixed Methods Pilot, Feasibility Study

**DOI:** 10.1155/2016/4723636

**Published:** 2016-10-19

**Authors:** Bernadette Bartlam, Trishna Rathod, Gillian Rowlands, Joanne Protheroe

**Affiliations:** ^1^Research Institute for Primary Care & Health Sciences, Keele University, Keele, UK; ^2^Institute of Public Health, Aarhus University, Aarhus, Denmark; ^3^Institute of Health/Society, Newcastle University, Newcastle upon Tyne, UK

## Abstract

This article reports a mixed methods process evaluation of a pilot feasibility randomised controlled trial comparing a Lay Health Trainer (LHT) intervention and usual care for those with poorly controlled Type 2 Diabetes Melitus (T2DM). Set in a deprived area in the UK, this research explores patient and health care practitioner (HCP) views on whether a structured interview between a patient and a Lay Health Trainer (LHT), for the purpose of developing a tailored self-management plan for patients, is acceptable and likely to change health behaviours. In doing so, it considers the implications for a future, randomised controlled trial (RCT). Participants were patients, LHTs delivering the intervention, service managers, and practice nurses recruiting patients to the study. Patients were purposively sampled on their responses to a baseline survey, and semistructured interviews were conducted within an exploratory thematic analysis framework. Findings indicate that the intervention is acceptable to patients and HCPs. However, LHTs found it challenging to work with older patients with long-term and/or complex conditions. In order to address this, given an ageing population and concomitant increases in those with such health needs, LHT training should develop skills working with these populations. The design of any future RCT intervention should take account of this.

## 1. Introduction

In the last 30 years the number of people in the world aged 60 or above has doubled from 378 million in 1980 to 759 million in 2010. It is projected to more than double again in the next 40 years, rising to two billion by 2050. In addition, the older population is itself ageing; currently, the “oldest old,” those aged 80 and above, represent 13% of the global population aged 60 and over; yet projections indicate that by 2050 that proportion will have grown to 20% [1]. Long-term conditions (LTCs) are more prevalent in older populations (58 percent of people over 60 compared to 14 percent under 40) and in more deprived groups (people in the poorest social class have a 60 percent higher prevalence than those in the richest social class and 30 percent more severity of disease). In the United Kingdom (UK), the number of people with more than one LTC is expected to rise from 1.9 million in 2008 to 2.9 million in 2018 and this increasing prevalence is considered to be one of the biggest challenges facing the National Health Service (NHS) [2]. In the light of the increasing pressures on health and social care created by an ageing population, the UK House of Lords recently called for an urgent revision of how care is delivered, arguing for a move toward more integrated, person-centred care [3].

Diabetes is an example of a LTC and the number of adults across the globe living with it has quadrupled since 1980 to 420 million people [4]. In the UK it is the fourth most prevalent LTC and has increased by 25 percent from 1,962,000 people in 2007 to 2,456,000 people in 2011 [2]. Factors driving this increase are largely lifestyle related, that is, obesity because of poor nutrition and a lack of physical activity [4]. Good clinical management of diabetes is critical as poor control can result in complications such as blindness, renal failure, neuropathy leading to impotence, and foot disorders that can result in amputation, stroke, and heart disease [5]. It may be that inadequate health literacy is a significant factor in the disproportionate burden of diabetes and diabetes-related complications in more socioeconomically disadvantaged populations [6]. Moreover, those with low health literacy have lower levels of good self-management of chronic disease, including poorer diabetes self-management [6, 7]. Health literacy can be defined as “the personal characteristics and social resources needed for individuals and communities to access, understand, appraise and use information and services to make decisions about health” [8].

As a part of the response to the growing number of people living with long-term conditions, a number of which relate to health behaviours, many countries have developed the role of health-related lifestyle advisors (HRLAs) [9]. In the UK the term Lay Health Trainer (LHT) has been adopted. LHTs are people living in the local community, intended to be demographically similar to those with whom they work, offering “support from next door” rather than “advice from on high” and taking a holistic approach. They are trained to a minimum of UK National Qualification Framework (NQF) level three in using techniques based on psychological and behavioural theories to help change behaviours (https://www.gov.uk/what-different-qualification-levels-mean/overview). The role emerged as a result of the UK Department of Health's “Choosing Health” public health White Paper [10], which had as its aim the reduction of health inequalities by targeting disadvantaged groups in order to increase healthy behaviours and create opportunities for employment and training. LHTs have been found to be effective in engaging with less heard groups and supporting them to make and maintain lifestyle changes. They aim to promote affordable and sociocultural relevant lifestyle advice within communities. However, they were not designed to work with specific health conditions and little work has explored their efficacy in chronic long-term condition management, such as diabetes [11, 12]. Nonetheless, Pennington and colleagues, in their systematic review of the effectiveness, cost-effectiveness, equity, and acceptability of different types of HRLA role, identified some evidence that lay-led self-management interventions can be both effectual and cost-effective [9].

## 2. The SHIPS Randomised Controlled Feasibility Pilot Trial

Given that self-management of Type 2 diabetes is dependent on healthy lifestyle choices, the Study of Health Trainer Improved Patient Self-management (SHIPS) was a randomised controlled feasibility pilot trial (RCT) to develop and then compare a LHT intervention to improve patient self-management with usual care for those with low health literacy and poorly controlled Type 2 diabetes mellitus (T2DM). Patients with HbA1c > 7.5 or 58 mmol/mol in at least the last two measures were eligible to be recruited from a socioeconomic disadvantaged population [13] (see Protheroe, Rathod, Bartlam, Rowlands, Richardson, and Reeves, this issue).

The feasibility, pilot RCT took place in a UK local government council authority funded health promotion service. This local service employed four LHTs to offer information and support to help individuals improve their lifestyle and general health, and it was overseen by two service managers. The service was located in a Victorian gate-lodge to a large public park, two miles from the town centre, with a bus every half an hour. The aim of not being located in an obvious health built environment, such as a clinic, was to emphasise supporting health and well-being from within the community. However, patients could be seen elsewhere if other venues were more convenient to them, including their local primary care centre or their own home.

The SHIPS study was a complex intervention and, in line with Medical Research Council guidance [14], the process evaluation reported here had three research objectives:

1. To explore if the intervention was considered acceptable to patients, health care practitioners (service managers and practice nurses), and LHTs
2. To explore whether patients, health care practitioners, managers, and LHTs considered the intervention likely to change health behaviours
3. To consider the implications of findings for any future RCT

SHIPS was reviewed and approved in the UK by the National Research Ethics Service Committee East Midlands-Derby 2: 11/EM/0294.

## 3. Methods

The qualitative methods reported here form part of a mixed methods approach to pilot RCTs, and both sampling and analysis were integrated with some of the baseline data from the pilot RCT (Table 1) [15].

Semistructured interviews (in person or by telephone) were carried out with patients in the intervention arm, the LHTs delivering the intervention, and the service managers and practice nurses (practice nurse is the term applied to nurses working as part of a primary care team within a family physician/general practice setting in the UK) recruiting patients to the study. The intervention consisted of a structured interview with the LHT, development of an individualised self-management plan with the identification of specific agreed goals, and up to three support telephone calls from the LHT for a maximum of six months. In addition, a self-management pamphlet on T2DM was developed which the LHTs gave to patients. This differed from usual LHT care, in which the LHTs in the study would normally work on a one-to-one basis with patients for up to 12 months. It also differed from usual practice in the UK, where LHTs generally work with patients over a six-to-twelve-week period [11].

### 3.1. Recruitment

**Figure 1:**
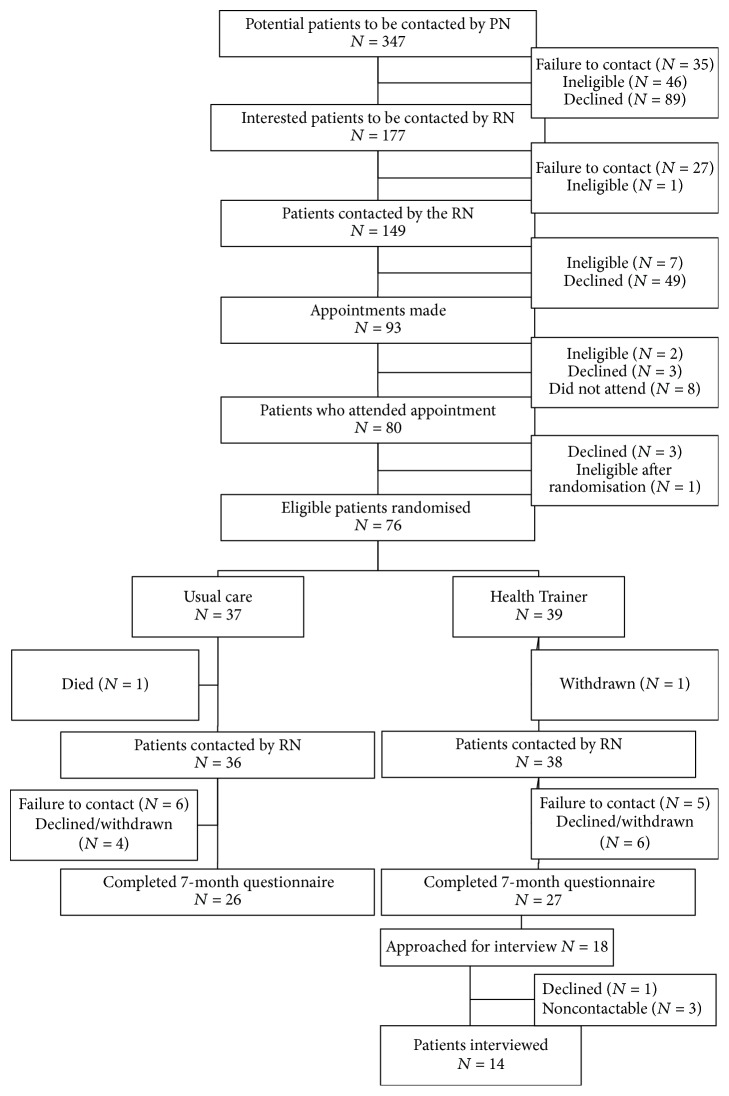
Patient recruitment process.

Recruitment of patients took place once the study team research nurse had completed the seven-month trial follow-up. This was to ensure sufficient time had lapsed for them to have had experience of the intervention. As part of this follow-up, they were asked to consent to further contact for the purposes of an interview exploring their views about the LHT service (Figure 1).

As previously mentioned, drawn from the baseline demographics in the pilot trial, a purposive sampling strategy based on an iterative analysis concurrent with data collection was used to ensure balance for factors likely to influence outcome such as diabetic control, length of time since diagnosis, age, and gender. Health literacy levels were also taken into account, using the Newest Vital Sign (NVS)UK. The NVS asks six questions based on a food label: a score of less than four is taken as indicating less than adequate health literacy [16]. In addition, scores on the Warwick-Edinburgh Mental Well-Being Scale (WEMWBS) were considered. The WEMWBS scores range from a minimum of 14 to a maximum of 70, with higher scores indicating better mental well-being. The WEMWBS population mean for England in 2012 was 52.4, with men scoring slightly higher than women [17]. Low WEMWBS scores are consistently associated with lower socioeconomic status [18].

The LHTs, service managers, and practice nurses involved in the trial were also invited to interview. Follow-up interviews also took place with the LHTs and service managers toward the end of the pilot trial, with the aim of checking if their views or experiences had changed since the initial interview. The practice nurses were interviewed after referring patients to the LHT service, so a follow-up interview was not deemed necessary. Since patients were interviewed once the seven-month follow-up with the research team nurse had taken place, this was considered sufficient time to capture change in views within that group.

All participants were offered a choice of interview format and, in case of a face-to-face interview, a choice of location. Patients received a Patient Information Leaflet (PIL) at the time of their seven-month follow-up, ahead of deciding whether to consent to contact about a possible interview. Having been contacted by the qualitative researcher, and agreeing to be interviewed, they were sent a further copy of the PIL ahead of the interview as an additional reminder and explanation. The information encouraged them to discuss the study with family or friends ahead of deciding whether or not to continue. The written information was developed in collaboration with the Patient and Public Involvement research user group within the Research Institute for Primary Care and Health Sciences at Keele University. This two-arm approach to informed consent was not considered necessary for the health professionals collaborating in the trial, who were familiar with the PIL/purpose of the interview study in order to answer any questions patients might have and who consequently received one set of information prior to their interview. Information to all interview participants emphasised that any quotes that might be used in publications would be anonymised, and names and personal details would not be used in such publications. Written consent was obtained from all participants.

### 3.2. Interviews

Interview topic guides were developed from the research objectives. Those for patients explored their overall health, the history of their diabetes, and their experience of the information they had received since diagnosis in terms of enabling them to understand and manage their condition. The interviews also explored their expectations, experiences, and views of working with their LHT and the extent to which they had changed their self-management as a result. The guide evolved in the light of emerging findings, which also informed the continuing sampling strategy. The questions to the LHTs, practice nurses, and service managers focused on their experiences of working with this particular patient population and what they considered the challenges and opportunities. They also explored aspects of practice and service provision—including the intervention—seen as useful, or not, in supporting behaviour change.

The steps outlined in the PIL on data anonymity and participant confidentiality were highlighted again before beginning the interview, and consent checked both at the start and end of the interview. The written information and consent forms for the LHTs, service managers, and practice nurses also highlighted both of these issues, providing a framework for discussion and checking. All discussions were digitally recorded and transcribed. All transcripts were anonymised and participants were given a unique numeric identifier. In addition, patients were given pseudonyms. Interviews lasted approximately half an hour, except for the dyadic interview with the two service managers which lasted one and a half hours, because of the more co-constructed nature of the discussion. Data collection with all four sets of participants took place between April and October, 2013.

### 3.3. Analysis

An exploratory thematic framework was adopted for the analysis, with emergent findings checked out in subsequent interviews across all four groups of participants in an iterative cycle. To maximize the benefits of being an interdisciplinary team, the two coders brought differing perspectives to bear on the data (Bernadette Bartlam, social science; Joanne Protheroe, family medicine). To ensure intercoder reliability, each independently coded a random selection of interviews as part of reaching agreement on the coding frame, which was then applied across the whole data set by Bernadette Bartlam, checking for consistencies and confounding cases [19–21].

## 4. Results 

### 4.1. Participants

In total, 24 participants were interviewed: 14 patients with poorly controlled T2DM, two service managers, four LHTs, and four practice nurses. Follow-up interviews also took place with three of the LHTs and one service manager, giving sufficient data to ensure that no issues had been overlooked. This gave a total of 28 interviews.

#### 4.1.1. Health Trainers

Three of the LHTs delivering the intervention were men, with one woman. One person had been in post six years, two for five years, and one for three years. All had undertaken the Royal Institute of Public Health “Understanding Health Improvement” course, NQF level two qualification, together with the City & Guilds Health Trainer course, NQF level three. In addition, they had all undertaken a variety of short courses on motivational interviewing and they all came from the local area. Three had previous backgrounds in health and fitness, and one had been a delivery driver. Their ages ranged from 26 to 34 years.

#### 4.1.2. Service Managers

One manager was a nurse with degree level education in public health who had been responsible for originally commissioning the LHT service. The other had been the day-to-day manager of the service since its inception in 2007 and had degree level education in Nutrition, Health, and Exercise, and in Voluntary and Third Sector Management.

#### 4.1.3. Practice Nurses

The four practice nurses recruiting patients to the study had been trained and working as primary care nurse specialists in diabetes for between six and eight years.

#### 4.1.4. Patients

Seventy-six patients were randomised into the pilot trial, 39 to the intervention arm. There was a follow-up rate at seven months of just under 70%, resulting in 27 patients available for invitation to interview. Twenty-two of these consented to recontact for a possible interview. The reasons for refusing were poor health of self or partner and having other commitments. Based on the sampling strategy, contact was attempted with 18 participants, three of whom were noncontactable—one person's phone number was “invalid” and it was not possible to contact the other two people, despite five attempts at different times on different days. One person that was contacted declined participation because of a recent bereavement (Figure 1).

Of the patients interviewed, six were women and eight were men. Ten participants were aged over 60 years; the age range was 43–86 years, with men being generally younger than the women (mean of 59 compared to 73 years). From the baseline data in the pilot RCT, the average length of time living with T2DM was 12 years. Although there was a considerable range in this (from one year to 25 years), the majority of participants (11) had lived with the condition for ten years or more. All participants were also living with at least one additional LTC, and the majority rated their own health as fair or good. However, with a mean score on the WEMWBS of 23, participants' mental well-being was very much lower than the UK population norm of 52.4 [17], with men scoring slightly higher overall. There was a spread of scores across the NVS, with the mean for women (2.5) being slightly lower than that for men (3) (Table 2). It is also worth noting that a number of participants who had low scores on the NVS self-reported their health as good or excellent.

### 4.2. Key Themes

Three key interrelated themes emerged from the analysis: health literacy and understanding of diabetes, responses and coping strategies, and motivation to change. In what follows we present details of these using illustrative quotations, before turning to look at the implications. Interviewer comments are in italics.

#### 4.2.1. Theme: Health Literacy and Understanding of Diabetes

The relationship between health literacy and people's understanding of their condition was immediately apparent, as this excerpt from the interview with Beth illustrates; she was an 86-year-old lady, diagnosed with diabetes for 12 years and with a low NVS score of two:

*I really don't feel it's as serious as they try to make out… The younger sister, she's abandoned all pills [for T2DM]. She doesn't have any.*

* And would you recommend the Health Trainer Service to her…? *

*Well, no, she has no problems. She does eat well… but she does drink a little bit too [laughs]… We can tell when she falls over that she's had a little bit too much [laughs], and she smokes… but she's healthy, you know.*

Similarly, Fred, a 72-year-old man with a low NVS score of one, who had lived with diabetes for 18 years, found it difficult to accept even general advice on health, as this excerpt shows:

*It says giving up smoking is one of the most positive things you can do to improve your health, right? Well, when I stopped smoking, just over two years ago, my diabetes became uncontrollable, so I disagree.*

This lack of health literacy was reflected in the interviews with the LHTs, as this account by LHT3 illustrates:

*One client was told by someone at the gym that he needed to be on a higher protein diet and cut out his carbohydrates, lose weight, and when I explained the Eat Well Plate to him, he wouldn't have it. *

Linked to this lack of clarity was a lack of understanding about the role of LHTs in supporting self-management of the condition, as these excerpts from the interview with LHT1 illustrates when reflecting on the people seen in the trial:

* They don't know who we are… they've had the condition for so many years and why haven't they addressed it before they've come to us? And they're so set in their ways now that they don't want to, there's quite a lot of resistance. *

Such lack of clarity could result in unrealistic expectations on the part of patients and of other health professions of what the service might offer, given the level of training and expertise amongst LHTs, as this interview with practice nurse 1 indicates:

*I think a Health Trainer would look at more like the whole person and the whole thing, whereas when we refer them to different services. They're either just looking at the weight loss, or they're just looking at smoking cessation, or they're just looking at alcohol, whereas there's a lot of other factors that come into the whole person.*

It was also apparent that the SHIPS pilot trial was recruiting patients who would not generally fall within the age range targeted by the service employing the LHTs, as Service Manager 2 clarifies:

*Just one thing that I noticed from this group from the SHIPS study that we don't tend to have with the people that we regularly support, is the age group. So when you said “Do you tend to deal with over 65?” and it might be so many, but after that we don't tend to have those older age groups… and so straightaway you've got issues around the fact that they've obviously had… the condition for a long time… the behaviour's so engrained… And it's a group that, although we deal with that group, it's not a large age category that's supported by health trainers usually.*

#### 4.2.2. Responses and Coping Strategies

Patients' lack of understanding of their condition was reflected in their self-management, as Beth's description of her diet illustrates:

* When you're old you can't possibly eat five portions of fruit and vegetables a day.*

Despite a score on the WEMWBS of 23.21, and even though she had multiple coexisting chronic health problems, Beth reported her own health as good.

The sense of already doing what was necessary to live well with diabetes was reflected throughout the interviews, as this excerpt from the interview with Tom, a 54-year-old man, diagnosed with diabetes for 14 years and with an NVS score of three, shows:

*The things they've got in the book [pamphlet] I eat anyway. I always have. I don't like MacDonald's, I can't be doing with that kind of rubbish*.

Jane, a 62-year-old woman living with diabetes for 20 years, with an NVS score of four, also felt she was managing well despite poor glycemic control:

*I do consider myself a bit of an expert because I've been diabetic for quite a while.*

However, this was not exclusively the case, as John, a 67-year-old man with a low NVS score of one and who had lived with diabetes for 15 years, illustrates when he responds to the question on the ways in which he found the LHT helpful:

*First of all I think what [LHT] done really, I started looking at what I eat because [LHT] explained everything… was very… not complicated, if you know what I mean?*

*Straightforward?*

*Straightforward and just said “If you want to control it you've got to do this. Without doing this, it won't work.” Simple as that. But very plainly told me what's the score.*

*Okay, and you found that helpful?*

*Very helpful, yes. *

Fred, too, despite his earlier scepticism over health advice, reported finding the consultation with the LHT helpful:

* [LHT] completely changed and broadened, in effect, what I was eating. And I feel a lot better as a result of that.*

The degree to which participants found the intervention helpful appears to be directly related to communication within the consultation.

#### 4.2.3. Motivation to Change

Motivation and capacity for change also emerged as an important factor, as Beth indicates when asked what she first though when the LHT was suggested:

*I think it was a bit of a waste of time, at my age, when I've had it for so long… I've had no problems.*

However, despite this she did feel that the intervention had brought some benefit:

* Do you recall setting goals with him?*

* Yes, when I knew I had to record what I was eating, it did make me eat better, because I had to put it down what I'd had, you know? I couldn't just say, “Oh, a couple of biscuits,” or something, you know, for a meal [yeah]. I did make the effort to eat properly while I was recording, you know.*

Whilst Jane recognised that her glycaemic control was poor, she reported not finding the LHT consultation helpful, echoing issues around long-established conditions and coping strategies and age:

* [LHT was] on about me doing more exercise than I do, and I do exercise [laughs] everyday. It's laughable really… I felt as though I was wasting his time. Then [LHT] was on about the food I was eating. Well, you go through this so many times. That's all they seem to think; because you've got a little bit of weight on, you need to lose weight. I hardly eat anything. I don't eat bread at all now. I don't buy crisps… And [LHT] says, “I can organise some cookery lessons for you.” I thought; “I'm 63, what do I want to do with cookery lessons at my age?”… So I said in the end, I says, “I think we're wasting our time here”.*

Jane's reluctance to engage with the LHT reflects her sense of herself as expert and also may be a reflection of her self-reported poor health status and her low score on the WEMWBS of 15.32. She clearly had complex health needs: throughout the interview she also spoke of her chronic heart condition, and the high impact that was having on her life. Shortly before participating in the trial she lost her mother and was finding it difficult to come to terms with that. Nonetheless, she spoke positively of the LHT as an individual: “He was excellent, really, it just didn't suit me…”

However, John, clearer in his understanding of his condition as a result of meeting with the LHT, reported being highly motivated to change, as this response to being asked whether he had identified particular goals shows:

* Lose weight [Laughing]. That's the number one… So I thought yes, I'll just follow what [LHT] said. And that's it. I lost two and a half stones. I put my control over diabetes into motion, really, and I figure that it's thanks to that LHT.*

John had one of the highest scores amongst participants on the WEMWBS, 28.13, and reported his health as good even though he too had coexisting LTCs.

Other factors, such as working conditions appeared to play a part in capacity for change, again illustrated by this response from Tom, a long-distance lorry driver, when asked about his views on the LHT service:

* He was nice lad everything, explaining like, this might help and that might help… It's all right now because I'm not working, I said, but as soon as I go back to work everything just goes out the window again… I sometimes miss my dinner-time tablets, because you start at daft times….*

* Did you just go and see him the once then?*

*Yes, because [LHT] said “due to your lifestyle there's not a lot you can do about it really”, you know? *

As the earlier excerpt illustrated, Tom felt that his diet was good. It was managing the medication and the complexity of his other health conditions that he found challenging, and which left him with a sense of being overwhelmed and unable to change. This was reflected in his WEMWBS score of 13.33, the lowest of all participants, and he reported his health as poor.

Fred highlights the importance of timing and early intervention after diagnosis, and the usefulness of the pamphlet the LHTs used to explain how to live well with diabetes

* So you've been living with it for a long time?*

* Oh, yeah, but not living with it correctly [laughs]. *

* So are you coming across stuff in [the pamphlet] that's new?*

* No. No, not really, no. [Pause] No, I mean, I am aware…*

* Of everything that's there?*

* Yeah.*

* But this would be something that you think would be useful for folk?*

* Oh, yes. If I'd have been given something like this in the early days, it would have been a much greater help than that which I received.*

* Right, in terms of understanding?*

* Yes, and making it plainer. You see, pictures are a better way of telling the story.*

* Yes, than just lots of words? *

* Yeah, because people tend to get a bit bored of lots of words, particularly if they're not presented well. *

Again, this was reflected in the interviews with the LHTs, as this quote from LHT4 indicates:

*I think the kind of patients that need to come in need to be people who want to change and are ready to change. It's perhaps better to get them when they've just been diagnosed. *

The challenge in how best to address the complexity with which patients could present, and their prioritisation of their needs, was also evident in the interviews with the LHTs:

* So when I was dealing with [patient name], there was lots of issues and a lot of the time was spent just listening and trying to help her deal with these issues… because her health and lifestyle was really poor… It was very hard to get her to engage in the topic of what we were looking at without her going off on a tangent. She kept apologising for the fact that she was doing all this talking, and she was talking about all this other stuff, which, it had some relevance, but it's not relevant for what we're trying to achieve. So she was aware that, you know, the study was to help with managing diabetes, and she wasn't allowing me to do that, and she wasn't able to engage in that.* (LHT3)

Finally, location of the LHT service was also something that emerged as an influencing factor on people's motivation and capacity to engage:

* I think location can make a difference to people, if it's hard for them to get to the service. That does sometimes figure as a factor in them not turning up for appointments.* (LHT4)

## 5. Discussion

People with long-term conditions can and do self-manage complex medical regimes every day including medicine taking, self-injecting, and dressing wounds as well as dealing with their many challenges of everyday living. They need help to have the confidence and knowledge to know what they can do effectively and safely for themselves and when to seek professional help. (Dr. Patricia Wilkie, President and Chairman National Association for Patient Participation [2].)

This study had three research objectives: first, to explore if the intervention was considered acceptable to patients with low health literacy and T2DM and to practitioners; second, to explore whether they considered if the intervention was likely to change health behaviours; and finally to consider any implications for a future main trial.

Given the complex characteristics of the study population, it is important to note that the SHIPS pilot trial results indicate that the intervention is feasible and should be carried forward into a main trial. Moreover, the pilot trial results indicate that the LHTs had a positive impact on the mental health of participants in the intervention arm compared to those in the control arm (see Protheroe et al., this issue, for full details of the pilot trial results). However, as the qualitative findings here indicate, and as to be expected, the picture is more nuanced than the trial findings alone suggest, with patients experiencing a range of responses in terms of the acceptability of the intervention and the likelihood of it resulting in behaviour change. Findings must be interpreted with caution given that participants were drawn from a small pilot study located in one specific area in the UK. Moreover, the data is cross-sectional and does not allow for follow-up over time. Finally, participants in this study all scored well below the population mean of the WEMEBS of 52.4, with a range of 13.33–35, despite most self-reporting their health as good. However, it is worth noting that older people have been found to be significantly less likely to partially or not respond to the tool [17], and more work is needed to establish its reliability in older populations and amongst those with low health literacy. Nonetheless, despite these limitations, these findings suggest that a full RCT intervention could be enhanced if attention were paid to a number of issues.

First, in keeping with the work of authors such as Carollo [22], relationships and communication emerged as critical. Even those patients who did not find the intervention helpful spoke of their experience of engaging with the LHT in positive terms, which itself is important in terms of likelihood of accessing support in the future. Whilst the LHTs and health care professionals in the study found the intervention acceptable, not all patients did so. This may be for a number of reasons. Key to engaging patients in behaviour change is clarity around roles and responsibilities. Whilst the LHTs and managers interviewed were very clear about the role, other health care professionals, and in particular patients, appeared less so, leading to unclear expectations for some patients. Greater promotion of the LHT service would improve patient and public understanding of what it can, and cannot, offer. With its emphasis on reaching those patients less likely to access services, careful thought needs to be given as to the ways in which such information is delivered [23]. In addition, there may be something in the title “Health Trainer” that may hold less appeal to older people who have been living with their condition(s) for protracted lengths of time. It may also be that, given the training and qualifications outlined here, the term “Lay Health Trainer” no longer reflects the original emphasis on amateur peer support from “next door.”

It was also clear from this work that LHTs appear to be effective for those patients who are already motivated to change health behaviours. However, they may be less effective with those patients who have a more established view of their condition, and those with complex health needs, for example, multiple LTCs, and those who are older [24]. Moreover, there remains a dearth of evidence around the relationship between adherence and older adults with low health literacy [25].

The LHT service in this study tended not to work with those over 65 years. In addition, most LHT services aim to recruit a high proportion of their staff from similar backgrounds to their clients [26] and it may be that the disparity in the average age of the LHTs in this study compared to participants (30 versus 64 years) had an impact on the potential therapeutic impact of the intervention. Given the ageing population and the concomitant increase in those growing older with more than one LTC, having the skills needed to engage such individuals will become more necessary. Such skills need to include an understanding of developmental ageing, in particular the challenges of later life [27, 28], as the individual psychosocial context within which any intervention is delivered.

Finally, location also emerged as an issue in this feasibility pilot RCT, with some LHTs and patients reporting challenges in accessibility, despite the efforts to offer a range of settings. Emphasising the message that a variety of consultation settings are available is something a future RCT should take account of. In addition, whilst this service was located in an urban environment, thought should also be given to how to best reach people living in rural areas, which are experiencing the fastest growth amongst older populations [13].

## 6. Conclusions

This work suggests that LHTs appear to be effective for those patients already motivated to change health behaviours but that they may be less effective with those who are older and have a more established view of their condition and how best to self-manage. However, recent systematic reviews indicate that, whilst interventions are potentially effective, there remains a paucity of evidence on this topic [25, 29, 30]. Further research is needed on the association between health literacy and general health behaviour and on the effectiveness of interventions such as those in the SHIPS pilot RCT. In particular, work is needed that can take into account the complexity of diverse populations, including issues such as environment, culture, gender, and life-course perspectives and which can allow for a longitudinal follow-up to evaluate the effectiveness of interventions. These qualitative findings highlight the importance of expanding LHT practice to develop skills around working with older populations. They also contribute to the argument for the inclusion of mixed methods, qualitative research in RCTs.

## Figures and Tables

**Table 1:** Summary of data.

	Interviews
Patients	14 one-to-one interviews (mean 30 mins)

Health Trainers	4 interviews 3 follow-up telephone interviews (mean 30 mins)

Service managers	2 x one dyadic interview (1 hr 50 mins) 1 follow-up telephone interview (24 mins)

Practice nurses	4 telephone interviews (mean 25 mins)

**Table 2:** Summary of patient participant characteristics.

	Women	Men	Overall
Gender (*N*)	6	8	14
Age (years) (mean, range)	73 (61, 86)	59 (43, 73)	70 (43, 86)
NVS (Mean)	2.5 (0, 5)	3 (0, 6)	0–6
Number of years living with T2DM (mean, range)	13 (1, 25)	10 (3, 18)	12 (1, 25)
Self-reported health			
Poor	2	1	3
Fair/good	4	5	9
Very good/excellent	0	2	2
WEMWBS score (mean, range)	22.5 (15.3, 32.6)	23.6 (13.3, 28.1)	23.0 (13.3–35.0)
